# Correction to ‘Organic matter removal from mother liquor of gas field wastewater by electro-Fenton process with the addition of H_2_O_2_: effect of initial pH’

**DOI:** 10.1098/rsos.200068

**Published:** 2020-02-05

**Authors:** Yan Wang, Hui-qiang Li, Li-ming Ren

*R. Soc. open sci.* **6**, 191304. (Published 11 December 2019). (doi:10.1098/rsos.191304)

This correction refers to errors in the labelling of the *y*-axis in figure 3. ENC and ELC were used in place of SEEC and SEPC. This has now been corrected.

**Figure 3.**
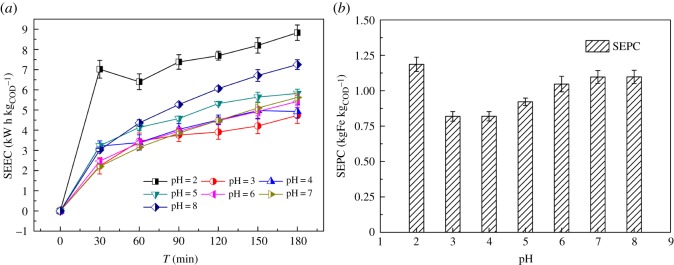
Effect of the initial pH on (*a*) SEEC in EF process, (*b*) SEPC in EF process. Conditions: 500 ml ML-GFW; I = 2 A; period reversal time: 10 min; H_2_O_2_: 7 ml/30 min; reaction time: 3 h.

